# Is private insurance enough to address barriers to accessing dental care? Findings from a Canadian population-based study

**DOI:** 10.1186/s12903-024-04271-0

**Published:** 2024-04-29

**Authors:** Mona Abdelrehim, Sonica Singhal

**Affiliations:** 1 Dental Public Health, Faculty of Dentistry, University of Toronto, Toronto, Canada; 2 Public Health Ontario, Toronto, Canada

**Keywords:** Cost barriers, Access to dental care, Self-perceived oral health, Dental care utilization, Dental insurance

## Abstract

**Background:**

In Canada, as in many other countries, private dental insurance addresses financial barriers to a great extent thereby facilitating access to dental care. That said, insurance does not guarantee affordability, as there are issues with the quality and level of coverage of insurance plans. As such, individuals facing barriers to dental care experience poorer oral health. Therefore, it is important to examine more keenly the socio-demographic attributes of people with private insurance to particularly identify those, who despite having insurance, face challenges in accessing dental care and experience poorer oral health.

**Methods:**

This study is a secondary data analysis of the most recent available cycle (2017-18) of the Canadian Community Health Survey (CCHS), a national cross-sectional survey. Univariate analysis was conducted to determine the characteristics of Ontarians with private insurance (*n* = 17,678 representing 6919,814 Ontarians)—bivariate analysis to explore their financial barriers to dental care, and how they perceive their oral health. Additionally, logistic regressions were conducted to identify relationships between covariates and outcome variables.

**Results:**

Analysis shows that the majority of those with private insurance do not experience cost barriers to dental care and perceive their oral health as good to excellent. However, specific populations, including those aged 20–39 years, and those earning less than $40,000, despite having private dental insurance, face significantly more cost barriers to access to care compared to their counterparts. Additionally, those with the lowest income (earning less than $20,000 annually) perceived their oral health as “fair to poor” more than those earning more. Adjusted estimates revealed that respondents aged 20–39 were six times more likely to report cost barriers to dental care and ten times more likely to visit the dentist only for emergencies than those aged 12–19. Additionally, those aged 40–59 were two times more likely to report poorer oral health status compared to those aged 12–19.

**Conclusion:**

Given the upcoming implementation of the Canadian Dental Care Plan, the results of this study can support in identifying vulnerable populations who currently are ineligible for the Plan but can be benefitted from the coverage.

## Introduction

Unlike universal coverage for general health care through public funds, dental care is a personal responsibility for the majority of Canadians except for medically necessary surgical-dental services delivered in publicly funded hospitals [1]. Approximately 32.4% of Canadians have no dental insurance and pay out-of-pocket when accessing dental care. Among those who are insured, 76.3% have insurance through their employer, 13.9% benefit from publicly funded insurance, and 9.7% purchase their own private insurance [2–4]. In 2018, Canadians’ out-of-pocket spending on dental care accounted for $6.3 billion; this amount represents 39% of the private dental care expenditure, which comes from those who have no insurance, purchase their own private insurance; and also from those who have employer-based insurance in the form of co-payment and deductibles [5].

With a large proportion of Canadians financing their dental care, cost becomes the predominant factor limiting access to care [6]. Previous studies have demonstrated that dental insurance, though it reduces financial barriers to dental care, does not completely eliminate those barriers [7, 8]. Insurance companies act as benefit carriers and reimburse patients based on their level of coverage, which in most cases is less than 100% [6]. Insurance plans differ in terms of the types and services covered, employer-employee percentage contributions, employee premiums, annual maximums, reimbursement rates, and wait periods before coverage begins [9].

Despite coverage, patients generally pay 20–50% of their dental care bill as a co-payment from out-of-pocket [10]. Accordingly, out-of-pocket payments for services not covered by insurance plans might pose an additional financial burden. Previous research revealed that out-of-pocket expenditures could represent a reasonable proxy of access; in other words, the more a household spends, the more difficult it may be to access dental care [11].

Overall, in the dental care market, although the proportion of private insurance has remained relatively stable over the last two decades, 63% in 2005 and 62% in 2018 [12], the quality of insurance has changed significantly; dental plans have limited the annual maximum, coverage of services, and have increased deductibles, co-payment, or co-insurance [13]. These changes have affected plan members’ satisfaction with their insurance plans; for example, in 1999, 73% of plan members reported that their health benefits plan met their needs “extremely” or “very well”, compared to 64% in 2020 [14–16]. Likewise, 59% of employees found the quality of their benefit plans to be “excellent” or “very good” in 2006, compared to 47% in 2020 [15, 16].

Data from the Canadian Community Health Survey (CCHS) 2018 cycle shows that 22.3% of Ontarians reported cost barriers to dental care, and among those who reported cost barriers to dental care, 64.1% had no insurance, 4.6% had public insurance, and 31.3% had private insurance [8]. In March 2022, the Canadian federal government announced plans to establish a national dental care program for low- and middle-income Canadians (those who have adjusted family net income of 90,000 or less) with no private insurance [17]. As we know that even with private insurance some people still face cost barriers to accessing dental care; restricting all Canadians with private insurance from enrolling in the national dental care program, simply by a dichotomization process, may leave many vulnerable falling through the cracks. It is important to identify who, despite of having private insurance, faces cost barriers to access dental care, and how they perceive their oral health. Hopefully, understanding the socio-demographic attributes of those, who despite having private insurance are not able to access dental care, would support the policy makers to have a more targeted and gradient approach to their eligibility criteria for the upcoming national dental care program.

## Methods

This study used the survey data from the CCHS, cycle 2017-18, from Ontario, Canada’s most populated province. The CCHS is a national population-based cross-sectional survey representing approximately 97% of Canadians. The survey collects information related to health status, healthcare utilization and health determinants for the Canadian population at the regional and provincial levels; it targets people aged 12 years or older, living in private dwellings from all 13 Canadian jurisdictions. Individuals living on reserves and other Indigenous settlements in the provinces, full-time members of the Canadian Forces and the institutionalized population do not constitute the sampling frame [18]. The oral health and dental care questionnaires are part of the optional content. This content was designed to address specific provincial-level needs; therefore, optional content questions were asked only in some provinces during each cycle and varied in content. In the latest cycle (2017-18), both oral health and dental care data were collected for the province of Ontario. Further information regarding the design and sampling characteristics of the CCHS can be found in the user guide [19].

The Public Use Microdata Files (PUMF) for the 2017-18 CCHS data were accessed online using the Survey Documentation and Analysis (SDA) online tool available through the University of Toronto library at the Computing in the Humanities and Social Sciences (CHASS) portal. No ethics review was sought for the study, as this is a secondary data analysis of anonymized data that contained no personal identifiers, nor was it linked to any other data source [20]. Additionally, this study was prepared following the Strengthening the Reporting of Observational Studies in Epidemiology (STROBE) cross-sectional reporting guidelines [21].

The population of interest for this study was those who have private dental insurance. Information regarding the availability and the type of dental insurance in the CCHS 2017-18 cycle was derived from the following questions “Do you have insurance or a government program that covers all or part of your dental expenses? and “Is it:….an employer-sponsored plan? …a provincial or territorial government program for children or seniors?… A private plan?… A government program for social service (welfare) clients?… A government program for First Nations and Inuit? The type of dental insurance variable was recoded and categorized into three groups “private insurance (employment-sponsored and self-purchased),” “government insurance,” and “no insurance.”

The next step was to assess two outcomes of interest among those with private insurance: their access to dental care; and oral health status. For the first outcome, access to dental care, measures included in this study were cost barriers to dental care, and frequency and type of dental visit. Measures were derived from the following two questions: (1) “In the past 12 months, have you avoided going to a dental professional because of the cost of dental care?” respondents answered “yes” or “no.” (2) “How often do you usually see a dental professional, such as a dentist, a dental hygienist or a denturist?” Respondents choose one of the following answers (a) more than once a year for check-ups or treatment; (b) about once a year for check-ups or treatment; (c) less than once a year for check-ups or treatment; (d) only for emergency care; (d) never.

Regarding the second outcome, oral health status, it is important to note that the CCHS does not provide any clinically assessed oral health measures, only self-reported ones. Therefore, this study examined oral health status through two variables: self-perceived oral health and satisfaction with teeth/denture appearance. Information on oral health status was obtained from the following two questions: (1) “In general, would you say the health of your mouth is…?” using a five-point scale from poor to excellent. This study grouped them into “good to excellent” and “fair to poor.” (2) How satisfied are you with the appearance of your teeth and/or dentures? using a five-point scale from very satisfied to very dissatisfied. In this study, the variable was grouped and categorized into three groups: “satisfied and very satisfied,” “neither satisfied nor dissatisfied,” and “dissatisfied and very dissatisfied.”

Covariates of interest included: age, sex, annual household income, the highest level of household education, employment status, marital status, culture/racial background, country of birth, length of time since immigration and health region. Age was categorized into five groups: “12–19,” “20–39,” “40–59,” “60–79,” and “80 and older”. Marital status was recoded and categorized into three groups “married/common law,” “widowed/divorced/separated,” and “single.” Education is indicated by the highest level of education of any member of the household and was dichotomized into “≤ secondary school graduation” and “> secondary school graduation.” The culture/racial background variable was dichotomized as “white” and “visible minority.” Similarly, country of birth was dichotomized as “Canada” and “other”, and length of time in Canada since immigration as “< 10 years” and “≥ 10 years.” Lastly, health region was categorized as “West,” “Central,” “Toronto,” “East,” and “North.”

The CCHS data was exported to a Microsoft Excel (© Microsoft 365 for Mac) worksheet and then imported into Stata v.17 software (© StataCorp: Release 17) for statistical analysis [22]. Missing data was excluded from this study. This study includes 17,678 Ontarians, and survey weights were applied during the data analysis to produce provincially representative results for a population of 6,919,814. Univariate and bivariate analyses were conducted to examine the sample characteristics and determine the characteristics of Ontarians with private dental insurance who reported cost barriers to dental care, visit the dentist only for emergencies, perceived their oral health as “fair to poor” and “dissatisfied and very dissatisfied” with their teeth/denture appearance. Then logistic regression was conducted to calculate unadjusted and adjusted odds ratios for identifying the risk indicators associated with reporting each outcome variable. Relevant independent variables were chosen for inclusion in the regression model, guided by previous literature, the significance level, and an assessment of multicollinearity among and between the variables (VIF < 3) [23]. The adjusted odds ratio, 95% confidence interval and p-value were reported for the variables in the regression model.

## Results

As per the CCHS 2017-18 cycle, approximately 62% (61.8, 95% CI: 60.1, 62.8) of Ontarians had private (employment-sponsored and/or self-purchased) dental insurance, which is the population of interest for this study (*n* = 17,678 representing 6919,814 Ontarians). Table 1 shows the baseline characteristics of Ontarians with private dental insurance in the 2017-18 CCHS cycle. Approximately 40% of the respondents with private dental insurance were aged 40–59 (95% CI: 37.9, 40.6). There was nearly an equal distribution of males and females. The majority of respondents were with a total annual household income of $80,000 (70.1, 95% CI: 68.9, 71.3), had more than high school education (87.10, 95% CI: 86.27, 87.89) and were married/common law (65.1, 95% CI: 63.8, 66.3). Around two-thirds of those with private dental insurance worked full-time (73.9, 95% CI: 72.7, 75.1), were white (72.0, 95% CI: 70.6, 73.4) and were born in Canada (69.8, 95% CI: 68.4, 71.1). 80% of those born outside Canada lived in Canada for ten years or more (95% CI: 77.0, 82.1). Lastly, residents of Central Ontario were more likely to have private dental insurance (29.5, 95% CI: 28.3, 30.7), followed by the West region (24.5, 95% CI: 23.5, 25.5) and the least was for those from the Northern region of Ontario (5.1, 95% CI: 4.8, 5.4).

**Table 1:** Basic demographic characteristics for Ontarians with private dental insurance, (2017-18, CCHS)

	Weighted (%)	95% CI
**Age**		
12–19 years	9.3	(8.6, 10.1)
20–39 years	32.5	(31.2, 33.7)
40–59 years	39.3	(37.9, 40.6)
60–79 years	17.3	(16.5, 18.2)
> 80 years	1.7	(1.5, 1.9)
**Sex**		
Male	49.5	(48.2, 50.9)
Female	50.5	(49.1, 51.8)
**Annual household income**		
No income or less than $20,000	1.8	(1.4, 2.2)
$20,000 to $39,999	5.1	(4.6, 5.7)
$40,000 to $59,999	11.1	(10.3, 12.0)
$60,000 to $79,999	11.9	(11.2, 12.8)
$80,000 or more	70.1	(68.9, 71.3)
**Highest level of household education**		
≤ secondary school graduation	12.9	(12.1, 13.7)
> secondary school graduation	87.1	(86.3, 87.9)
**Employment status**		
Full-time employed	73.9	(72.7, 75.1)
Part-time employed	9.9	(9.1, 10.8)
Unemployed	16.2	(15.2, 17.1)
**Marital Status**		
Married/ Common law	65.1	(63.8, 66.3)
Widowed/divorced/ separated	8.2	(7.6, 8.8)
Single	26.7	(25.5, 27.9)
**Cultural/racial background**		
White	72.0	(70.6, 73.4)
Visible minority	28.0	(26.6, 29.4)
**Country of birth**		
Canada	69.8	(68.4, 71.1)
Other	30.2	(28.9, 31.6)
**Length of time since immigration**		
< 10 years	20.3	(17.9, 23.1)
≥ 10 years	79.7	(77.0, 82.1)
**Health regions**		
West	24.5	(23.5, 25.5)
Central	29.5	(28.3, 30.7)
Toronto	19.9	(18.1, 21.4)
East	21.0	(20.1, 21.9)
North	5.1	(4.8, 5.4)

There are 17,678 individuals sampled representing 6919,814 Ontarians

## Access to care

Table 2 demonstrates the proportions of Ontarians with private dental insurance who faced cost barriers to dental care and visited the dentist only for emergency. The results show that 11.5% (95% CI: 10.7, 12.7) of Ontarians with private dental insurance reported cost barriers to dental care. The proportion distribution across different demographics was wide though ranging from 2.4% (95% CI: 1.5, 3.6) for 12–19 years of age to 22.9% (95% CI: 18.6, 27.8) for those with $20,000 to $39,999 annual household income. Statistically significant differences in proportions were observed among age groups, income levels, employment statuses, education levels, racial backgrounds, and Canadian/non-Canadian born.

Regarding the type of dental visit, 5.7% (95% CI: 5.2, 6.4) of Ontarians with private dental insurance visited the dentist only for emergencies. Based on socio-demographic attributes, it ranged from 0.9% (95% CI: 0.5, 1.7) among 12–19 year olds to 17% (95% CI: 8.7, 30.5) for those whose annual household income was less than $20,000. Statistically significant differences in proportions were observed among age groups, income levels, and education levels, and between sexes, and by length of time since immigration.

**Table 2:** Access to dental care among those with private dental insurance in Ontario according to their characteristics, (2017-18, CCHS)

	Cost barriers to dental care* Weighted % (95%CI)		Visited only for emergency** Weighted % (95%CI)	
All	11.5 (10.7, 12.7)		5.7 (5.2, 6.4)	
**Age**				
12–19 years	2.4 (1.5, 3.6)	P value = < 0.0001	0.9 (0.5, 1.7)	***P*** **-value** < 0.0001
20–39 years	16.7 (14.9, 18.6)		7.1 (5.9, 8.5)	
40–59 years	11.0 (9.7, 12.5)		5.2 (4.4, 6.1)	
60–79 years	8.1 (6.9, 9.6)		6.3 (5.2, 7.6)	
> 80 years	7.0 (4.1, 11.6)		14.2 (10.4, 19.2)	
**Sex**				
Male	10.8 (9.6, 12.2)	P value = 0.1243	6.5 (5.6, 7.5)	P value = 0.0017
Female	12.2 (11.1, 13.4)		5.0 (4.3, 5.8)	
**Annual household income**				
No income or less than $20,000	21.0 (14.6, 29.2)	P value = < 0.001	17.0 (8.7, 30.5)	P value = < 0.001
$20,000 to $39,999	22.9 (18.6, 27.8)		13.0 (9.9, 17.0)	
$40,000 to $59,999	10.0 (15.1, 21.4)		8.6 (6.9, 10.7)	
$60,000 to $79,999	14.5 (12.3, 17.0)		7.6 (6.2, 9.4)	
$80,000 or more	8.9 (7.9, 9.9)		4.2 (3.6, 4.9)	
**Highest level of household education**				
≤ secondary school graduation	14.5 (12.1, 17.2)	P value = 0.0043	11.2 (9.4, 13.3)	P value = < 0.001
> secondary school graduation	10.9 (10.0, 11.9)		4.8 (4.2, 5.5)	
**Employment status**				
Full-time employed	12.7 (11.6, 13.9)	P value = 0.0372	6.1 (5.3, 7.0)	P value = 0.2121
Part-time employed	12.8 (10.1, 16.1)		4.6 (3.1, 6.8)	
Unemployed	9.5 (7.7, 11.6)		6.1 (4.8, 7.8)	
**Marital Status**				
Married/ Common law	11.4 (10.4, 12.5)	P value = 0.4490	5.8 (5.1, 6.7)	P value = 0.1354
Widowed/divorced/ separated	13.2 (10.9, 15.8)		8.5 (6.6, 10.8)	
Single	11.2 (9.7, 13.0)		4.7 (3.7, 5.9)	
**Cultural/racial background**				
White	10.6 (9.7, 11.5)	P value = 0.0028	5.4 (4.9, 6.1)	P value = 0.1331
Visible minority	13.9 (11.9, 16.1)		6.4 (5.0, 8.1)	
**Country of birth**				
Canada	10.8 (9.9, 11.8)	P value = 0.0104	5.4 (4.8, 6.0)	P value = 0.2490
Other	13.4 (11.6, 15.5)		6.6 (5.3, 8.2)	
**Length of time since immigration**				
< 10 years	16.5 (12.1, 22.1)	P value = 0.2047	10.9 (6.9, 16.8)	P value = 0.0047
≥ 10 years	13.1 (11.0, 15.5)		5.2 (4.0, 6.6)	
**Health regions**				
West	11.1 (9.8, 12.6)	P value = 0.8092	6.3 (5.3, 7.5)	P value = 0.1732
Central	11.8 (10.2, 13.6)		5.0 (4.0, 6.1)	
Toronto	11.6 (9.3, 14.5)		5.6 (4.0, 7.7)	
East	11.8 (10.3, 13.5)		5.7 (4.7, 7.0)	
North	9.9 (8.3, 11.7)		8.3 (6.8, 10.0)	

*There are 17,668 individuals sampled representing 6914,920 Ontarians

**There are 17,655 individuals sampled representing 6913,075 Ontarians

Regarding the type of dental visit, 5.7% (95% CI: 5.2, 6.4) of Ontarians with private dental insurance visited the dentist only for emergencies. Based on socio-demographic attributes, it ranged from 0.9% (95% CI: 0.5, 1.7) among 12–19 year olds to 17% (95% CI: 8.7, 30.5) for those whose annual household income was less than $20,000. Statistically significant differences in proportions were observed among age groups, income levels, and education levels, and between sexes, and by length of time since immigration.

Table 3 illustrates the adjusted odds of Ontarians reporting cost barriers to dental care and visiting the dentist only for emergencies. For cost barriers to dental care, all age groups, 20–39, 40–59, and 60–79 years, reported higher odds of cost barriers compared to 12–19 years of age, with the highest disadvantage reported by 20–39 year olds, at 6.6 (95% CI: 3.6, 12.2). By income, all income groups were more likely to report cost barriers to access to care compared to those earning $80,000 or more annually, with the highest disadvantage reported by those earning $20,000 to $39,000 annually at 3.5 (95% CI: 5.6, 4.7). For visiting a dentist only for emergency, again all age groups, 20–39, 40–59, and 60–79 year olds were at higher odds than 12–19 year olds; however, the magnitude was more pronounced for all age groups. The highest disadvantage was though reported by 20–39 year olds, at 10.5 (95% CI: 4.1, 26.7). By income, all income groups were more likely to visit a dentist only for emergency compared to those earning 80,000 or more, with the highest disadvantage reported by those earning less than $20,000 annually at 3.8 (95% CI: 1.4, 10.0). Males and people with less than secondary school education were also reported to be more likely to visit a dentist only for emergency.

**Table 3:** Adjusted odds ratio for reporting cost barriers to dental care and visiting the dentist only for emergencies among Ontarians with private dental insurance, (2017-18, CCHS)

Independent variables	Cost barriers to dental care		Visited only for emergency	
	Adjusted OR (95% CI)	P-value	Adjusted OR (95% CI)	P-value
**Age (12–19 as a reference group)**				
20–39 years	6.6 (3.6, 12.2)	< 0.001	10.5 (4.1, 26.7)	< 0.001
40–59 years	4.3 (2.3, 7.9)	< 0.001	7.3 (2.9, 18.3)	< 0.001
60–79 years	2.7 (1.5, 4.9)	0.002	8.4 (3.3, 21.7)	< 0.001
> 80 years	N/A	N/A	N/A	N/A
**Sex female as a reference group)**				
Male	0.9 (0.8, 1.1)	0.460	1.7 (1.3, 2.1)	< 0.001
**Annual household income ($80,000 or more as a reference group)**				
No income or less than $20,000	2.6 (1.5, 4.5)	0.001	3.8 (1.4, 10.0)	0.007
$20,000 to $39,999	3.5 (5.6, 4.7)	< 0.001	2.9 (2.0, 4.20)	< 0.001
$40,000 to $59,999	2.4 (1.8, 3.2)	< 0.001	1.8 (1.3, 2.5)	0.001
$60,000 to $79,999	1.7 (1.3, 2.1)	< 0.001	1.8 (1.3, 2.4)	< 0.001
**Highest level of household education (> secondary school graduation)**				
≤ secondary school graduation	1.2 (0.9, 1.5)	0.226	1.9 (1.4, 2.5)	< 0.001
**Employment status (unemployed as a reference group)**				
Full-time employed	1.1 (0.8, 1.5)	0.695	1.0 (0.7, 1.3)	0.808
Part-time employed	1.2 (0.8, 1.8)	0.301	0.9 (0.5, 1.4)	0.523

## Oral health status

Table 4 demonstrates the proportions of Ontarians with private dental insurance who perceived their oral health as “fair to poor” and were “dissatisfied and very dissatisfied” with their teeth/denture appearance. The results show that 7.7% (95% CI: 7.1, 8.4) of Ontarians with private dental insurance perceived their oral health as “fair to poor”. Based on socio-demographic attributes, it ranged from 4.3% (95% CI: 3.2, 5.8) among 12–19 year olds to 15.2% (95% CI: 10.4, 21.6) for those with less than $20,000 annual household income. Statistically significant differences in proportions were observed among age groups, income levels, marital statuses, and geographical locations, and between sexes, and education levels.

In terms of teeth/denture appearance, 5.3% (95% CI: 4.8, 5.9) of Ontarians with private dental insurance were “dissatisfied and very dissatisfied”. The distribution was again varied, ranging from 3% (95% CI: 2.1, 4.1) among people living in Toronto to 9.4% (95% CI: 5.9, 14.7) for those with less than $20,000 annual household income. Similar to oral health status, statistically significant differences in proportions were observed among age groups, income levels, marital statuses, and geographical locations, and between sexes, and education levels. In addition, people who immigrated more than 10 years ago were more “dissatisfied and very dissatisfied” with their teeth/denture appearance than their counterparts.

**Table 4:** Oral health status of those with private dental insurance in Ontario according to their characteristics, (2017-18, CCHS)

	“Fair to poor” perceived oral health* Weighted % (95%CI)		“Dissatisfied and very Dissatisfied” with teeth/denture appearance** Weighted % (95%CI)	
All	7.7 (7.1, 8.4)		5.3 (4.8, 5.9)	
**Age**				
12–19 years	4.3 (3.2, 5.8)	P value = < 0.001	3.4 (2.1, 5.4)	P value = 0.0004
20–39 years	6.6 (5.6, 7.8)		4.3 (3.5, 5.1)	
40–59 years	8.8 (7.6, 10.0)		6.4 (5.4, 7.6)	
60–79 years	9.0 (7.7, 10.5)		5.7 (4.7, 6.8)	
> 80 years	9.4 (6.3, 13.9)		6.8 (4.4, 10.2)	
**Sex**				
Male	8.7 (7.8, 9.7)	P value = 0.0021	5.2 (4.5, 6.1)	P value = 0.0288
Female	6.7 (5.8, 7.6)		5.4 (4.7, 6.2)	
**Annual household income**				
No income or less than $20,000	15.2 (10.4, 21.6)	P value = < 0.001	9.4 (5.9, 14.7)	P value = 0.0017
$20,000 to $39,999	11.2 (8.7, 14.2)		8.2 (6.3, 12.5)	
$40,000 to $59,999	11.0 (9.1, 13.4)		6.5 (5.3, 8.1)	
$60,000 to $79,999	7.3 (6.0, 8.9)		5.9 (4.8, 7.2)	
$80,000 or more	6.8 (6.0, 7.6)		4.7 (4.0, 5.4)	
**Highest level of household education**				
≤ secondary school graduation	12.6 (10.7, 14.8)	P value = < 0.001	9.1 (7.2, 11.3)	P value = < 0.001
> secondary school graduation	6.9 (6.2, 7.6)		4.8 (4.3, 5.4)	
**Employment status**				
Full-time employed	7.6 (6.8, 8.5)	P value = 0.1318	5.2 (4.5, 5.9)	P value = 0.0917
Part-time employed	6.9 (5.0, 9.5)		6.0 (4.0, 8.9)	
Unemployed	9.4 (7.8, 11.3)		6.9 (5.5, 8.7)	
**Marital Status**				
Married/ Common law	7.9 (7.1, 8.8)	P value = 0.0031	5.5 (4.8, 6.3)	P value = 0.0390
Widowed/divorced/ separated	10.3 (8.4, 12.5)		7.4 (5.9, 9.2)	
Single	6.4 (5.4, 7.5)		4.2 (3.4, 5.2)	
**Cultural/racial background**				
White	7.5 (6.9, 8.3)	P value = 0.9723	5.4 (4.9, 6.1)	P value = 0.6660
Visible minority	7.5 (6.1, 9.2)		4.8 (3.6, 6.3)	
**Country of birth**				
Canada	7.3 (6.6, 8.0)	P value = 0.1367	5.5 (4.9, 6.1)	P value = 0.5534
Other	8.5 (7.1, 10.1)		5.0 (4.0, 6.4)	
**Length of time since immigration**				
< 10 years	6.7 (4.2, 10.5)	P value = 0.1643	3.3 (2.0, 5.5)	P value = 0.0118
≥ 10 years	9.4 (7.8, 11.4)		5.6 (4.2, 7.3)	
**Health regions**				
West	8.5 (7.8, 10.1)	P value = 0.0468	5.9 (5.0, 6.8)	P value = 0.0070
Central	7.9 (6.7, 9.2)		5.7 (4.5, 7.1)	
Toronto	6.2 (4.6, 8.3)		3.0 (2.1, 4.1)	
East	7.0 (5.8, 8.4)		6.2 (5.1, 7.5)	
North	9.7 (8.1, 11.5)		6.4 (5.1, 7.9)	

*There are 17,667 individuals sampled representing 6917,039 Ontarians

**There are 17,658 individuals sampled representing 6913,824 Ontarians

Table 5 illustrates the adjusted odds ratios for reporting “fair to poor” oral health and perceiving “dissatisfied and very dissatisfied” with their teeth/denture appearance. For “fair to poor” oral health, all age groups were at higher odds of perceiving “fair to poor” oral health compared to those 12–19 years of age with the highest disadvantage reported by 40–59 year olds, at 2.6 (95% CI: 1.6, 4.2). All annual household income levels up to $59,999, reported higher odds of perceiving “fair to poor” oral health compared to those earning $80,000 or more. Males and people with less than secondary school education were also reported to be more likely to perceive “fair to poor” oral health compared to their counterparts.

For being “dissatisfied and very dissatisfied” with their teeth/denture appearance, only 40–59 year olds were at higher odds, at 2.3 (95% CI: 1.0, 5.4), compared to 12–19 year olds. The only other attribute which was statistically significant was education; people with less than secondary school education were more likely, at 1.7 (95% CI: 1.2, 2.5), to be “dissatisfied and very dissatisfied” with their teeth/denture appearance compared to their counterparts.

**Table 5:** Adjusted odds ratio for reporting “fair to poor” oral health and “dissatisfied and very dissatisfied” with teeth/dentures appearance among Ontarians with private dental insurance, (2017-18, CCHS)

Independent variables	“Fair to poor” perceived oral health		“Dissatisfied and very dissatisfied” with teeth/denture appearance	
	Adjusted OR (95% CI)	P-value	Adjusted OR (95% CI)	P-value
**Age (12–19 as a reference group)**				
20–39 years	1.8 (1.1, 3.1)	0.025	1.5 (0.6, 3.5)	0.367
40–59 years	2.6 (1.6, 4.2)	< 0.001	2.3 (1.0, 5.4)	0.066
60–79 years	2.1 (1.3, 3.5)	0.004	1.8 (0.7, 4.3)	0.218
> 80 years	N/A	N/A	N/A	N/A
**Sex (female as a reference group)**				
Male	1.4 (1.1, 1.7)	0.003	1.1 (0.9, 1.4)	0.454
**Annual household income ($80,000 or more as a reference group)**				
No income or less than $20,000	2.3 (1.4, 3.8)	0.002	1.7 (0.9, 3.3)	0.094
$20,000 to $39,999	1.7 (1.3, 2.4)	< 0.001	1.8 (1.1, 3.1)	0.023
$40,000 to $59,999	1.6 (1.2, 2.1)	0.003	1.3 (1.0, 1.1)	0.088
$60,000 to $79,999	1.0 (0.8, 1.4)	0.830	1.2 (0.9, 1.7)	0.200
**Highest level of household education (> secondary school graduation)**				
≤ secondary school graduation	1.7 (1.3, 2.2)	< 0.001	1.7 (1.2, 2.5)	0.003
**Employment status (full-time employed as a reference group)**				
Part-time employed	1.1 (0.7, 1.6)	0.654	1.3 (0.8, 2.1)	0.293
Unemployed	1.3 (1.0, 1.8)	0.094	1.3 (0.9, 1.9)	0.097

## Discussion

Previous studies have primarily focused on individuals lacking private dental insurance, emphasizing the significance of insurance in accessing dental care. This study is the first, to the best of our knowledge, to specifically examine individuals with private insurance. Dental insurance stands out as a crucial facilitator for accessing oral health care. However, even among those with private insurance, some individuals encounter barriers to care. It is important to recognize that our study’s scope does not aim to assess whether private insurance mediates the relationship between socioeconomic status and access to care or oral health status. Instead, our study attempted to understand the characteristics of those individuals, who despite having private insurance experience financial barriers in accessing dental care and report poor oral health status.

As per the latest available statistics from 2017 to 18, approximately 62% of Ontarians have private insurance for dental care. Of these, almost 1 in 9 face barriers to dental care. However, this proportion changes by socio-demographic attributes. For example, among those earning less than 40,000, one in five faced barriers to access care vs. those earning more than $80,000, it was one in 11. As such, only 7% of the respondents, who had private insurance, were earning below $40,000, which is understandable as past studies have shown that income and insurance are correlated [24]. By age, the difference was further larger; for those aged 20–39 years, one in six face barriers and for those aged 12–19 years, it was one in 42. The data shows that 32% of those having private insurance are of the age 20–39 years; making us realize that a substantial Ontario population faces barriers to care. In terms of oral health, approximately 8% of those having private insurance perceive it as “fair to poor”; nonetheless, among those earning less than $20,000, 15% perceive their oral health as “fair to poor.”

Overall, this study shows that majority of those who have private insurance have good to excellent oral health and do not face cost barriers to access to care. However, there are certain specific populations, such as those aged 20–39 years, and earning less than $40,000, who face significantly more cost barriers to access to care. Also, those who are at the bottom of the barrel, earning less than $20,000 annually, perceive their oral health as “fair to poor” more than their counterparts. Despite having private insurance, they might experience financial barriers in covering the co-payments required for their dental visit. In addition, those with lower incomes might have unmet dental needs because they are unable to afford co-payments for dental treatment, leading them to perceive their oral health as “fair to poor” more than their counterparts. These results show that though private insurance is an important facilitator to access dental care, the wealth-health gradient [25–27] cannot be ignored as private insurance facilitates access only above a certain income gradient.

Enhancing the quality of dental insurance coverage in Canada is crucial. Changes such as limiting yearly maximums, restricting basket of services, and higher proportions of co-payments have adversely affected the quality of these plans [13, 28]. Furthermore, a notable gap between premiums collected and benefits paid by insurance companies has been observed in recent years [9]. In the absence of adequate coverage, individuals are compelled to spend more money out-of-pocket if they ultimately decide to seek care. Evidence indicates that the higher the out-of-pocket expenses, the more challenging it may be to access care, leading to a greater likelihood of reporting unfavourable oral health conditions [11]– a finding consistent with our results for Ontarians aged 20–39.

Our study’s strengths include a large sample size, the use of sample weights in the analysis, enabling population-level estimations in Ontario, the most populated province, and the use of data from the most recent CCHS cycle. However, the study also has limitations. Firstly, being a secondary data analysis of a national survey, we cannot detect or correct data entry errors from the original survey. Secondly, as the CCHS is cross-sectional, our study focuses on hypothesis generation rather than hypothesis testing, allowing only associations and no causal relationships to be inferred. Thirdly, outcome variables rely on respondents’ reporting of behaviour, potentially leading to socially desirable answers. Fourthly, the use of single-item questions in the CCHS may affect the validity of the responses compared to multiple-item questions. Lastly, our findings may be underestimated and cannot be generalized due to the exclusion of people living on reserves, full-time members of the Canadian Forces, the institutionalized population, and children aged 12–17 living in foster care.

## Conclusion

Dental insurance, although it reduces financial barriers to dental care to a great extent, does not guarantee affordability. Certain populations with private dental insurance, based on their socio-demographics, still experience financial barriers to dental care and report inferior oral health, particularly individuals aged 20–39 years old and those with annual household incomes less than $40,000. As the Canadian Dental Care Plan is currently under works, the results of this study have the potential to support the upcoming program by helping in identifying those vulnerable populations, who are not considered eligible yet and may fall through the cracks, irrespective of the new safety nets. Customizing the program based on this insight can facilitate a progressive approach that covers all those who could benefit from this new initiative.

## Data Availability

The Public Use Microdata Files (PUMF) for the 2017-18 CCHS data were accessed online using the Survey Documentation and Analysis (SDA) online tool available through the University of Toronto library at the Computing in the Humanities and Social Sciences (CHASS) portal. Requests and further information on accessing the dataset can be obtained here: https://mdl.library.utoronto.ca/research/help.

## Abbreviations

CCHS: The Canadian Community Health Survey
PUMF: Public Use Microdata Files
SDA: Survey Documentation and Analysis
CHASS: Computing in the Humanities and Social Sciences
OR: Odds ratio
CI: Confidence interval
